# Examining the effectiveness of telemonitoring with routinely acquired blood pressure data in primary care: challenges in the statistical analysis

**DOI:** 10.1186/s12874-021-01219-8

**Published:** 2021-02-10

**Authors:** Richard A. Parker, Paul Padfield, Janet Hanley, Hilary Pinnock, John Kennedy, Andrew Stoddart, Vicky Hammersley, Aziz Sheikh, Brian McKinstry

**Affiliations:** 1 Usher Institute, University of Edinburgh, Edinburgh, UK; 2 School of Health and Social Care. Edinburgh Napier University, Edinburgh, UK; 3 Edinburgh Medical School, University of Edinburgh, Edinburgh, UK

**Keywords:** Routine data, Implementation study, Quasi-experimental, Telemonitoring, Blood pressure control, Hypertension, End digit preference

## Abstract

**Background:**

Scale-up BP was a quasi-experimental implementation study, following a successful randomised controlled trial of the roll-out of telemonitoring in primary care across Lothian, Scotland. Our primary objective was to assess the effect of telemonitoring on blood pressure (BP) control using routinely collected data. Telemonitored systolic and diastolic BP were compared with surgery BP measurements from patients not using telemonitoring (comparator patients). The statistical analysis and interpretation of findings was challenging due to the broad range of biases potentially influencing the results, including differences in the frequency of readings, ‘white coat effect’, end digit preference, and missing data.

**Methods:**

Four different statistical methods were employed in order to minimise the impact of these biases on the comparison between telemonitoring and comparator groups. These methods were “standardisation with stratification”, “standardisation with matching”, “regression adjustment for propensity score” and “random coefficient modelling”. The first three methods standardised the groups so that all participants provided exactly two measurements at baseline and 6–12 months follow-up prior to analysis. The fourth analysis used linear mixed modelling based on all available data.

**Results:**

The standardisation with stratification analysis showed a significantly lower systolic BP in telemonitoring patients at 6–12 months follow-up (-4.06, 95% CI -6.30 to -1.82, *p* < 0.001) for patients with systolic BP below 135 at baseline. For the standardisation with matching and regression adjustment for propensity score analyses, systolic BP was significantly lower overall (− 5.96, 95% CI -8.36 to − 3.55 , *p* < 0.001) and (− 3.73, 95% CI− 5.34 to − 2.13, p < 0.001) respectively, even after assuming that − 5 of the difference was due to ‘white coat effect’. For the random coefficient modelling, the improvement in systolic BP was estimated to be -3.37 (95% CI -5.41 to -1.33 , *p* < 0.001) after 1 year.

**Conclusions:**

The four analyses provide additional evidence for the effectiveness of telemonitoring in controlling BP in routine primary care. The random coefficient analysis is particularly recommended due to its ability to utilise all available data. However, adjusting for the complex array of biases was difficult. Researchers should appreciate the potential for bias in implementation studies and seek to acquire a detailed understanding of the study context in order to design appropriate analytical approaches.

**Supplementary Information:**

The online version contains supplementary material available at 10.1186/s12874-021-01219-8.

## Background

Implementation studies enable the evaluation of research interventions in a real-world context, for example, in routine primary care. When combined with the collection of longitudinal data from electronic health records or data which are otherwise routinely acquired, these evaluation studies are not only data rich in terms of the information they provide, but are often based on patient populations that are highly generalisable and representative of the target population [1, 2]. Such studies provide great opportunities, but also great challenges, many of which are outlined in this paper.

Since 2015, in Lothian (south east Scotland), the Scale-Up BP implementation project has been using telemonitoring to monitor blood pressure (BP) in people with previously diagnosed hypertension. The telemonitoring system was designed based on the findings of the Health Impact of nurse-led Telemetry Services (HITS) randomised controlled trial [3] and subsequent research to explore barriers to implementation [4]. Participants used an electronic oscillometric sphygmomanometer to measure BP and then submitted BP readings via their own mobile phone using a low-cost third-party text-based telemonitoring system procured by the Scottish Government (Florence) [5]. These patient-generated BP readings were stored in a central server and made available to practices via an Internet link. Summaries of BP data were then displayed in the primary care data management system, Docman, at intervals chosen by the clinicians. Patients were informed by automated text responses if submitted readings were low, normal, high, or very high and were advised to follow a written action plan with respect to contacting their practice either routinely or urgently as appropriate. Full details about the system and the overall Scale-up BP project are provided elsewhere [6]. The Scottish Government’s Technology Enabled Care (TEC) fund [7] financed the third-party telemonitoring service, the development of the software to link it with GP systems using Docman, supported facilitators to visit/train practices, and purchased sphygmomanometers for loan to patients.

Although randomised controlled trials [8] have shown the effectiveness of telemonitoring in monitoring BP, the effectiveness and impact when the system is provided as a routine approach to care in general practice is uncertain. The Scale-up BP evaluation study aimed to use routinely acquired data and outcomes (including BP readings) extracted from GP records to evaluate the impact and acceptability of telemonitoring in this population. Eight practices were purposely chosen to be representative of all practices in Lothian such that they represented a range of sizes, levels of deprivation, and length of time since first adopting the system. Systolic and diastolic BP values from patients in the eight practices who used the telemonitoring system were then compared with BP values from patients who did not use the telemonitoring system from the same practices. The results of this evaluation study are published elsewhere [6], but in that paper we did not include a detailed comparison of BP between intervention and comparator groups.

In this article we present the results of an in-depth analysis employing a range of methods to investigate if telemonitoring improves BP control when routinely implemented at scale, while illustrating some of the challenges involved with evaluating effectiveness in a quasi-experimental study involving routinely acquired data.

Comparison of surgery readings with home telemonitored readings was challenging in this context for eight main reasons which are outlined in Table 1.

**Table 1:** Description of the challenges and potential biases faced in this study

(1) Non-randomised design	Patients were not randomised as this was a scheduled implementation of an evidence-based intervention [6]. In some practices almost all patients on the practice hypertension register were offered the intervention, but other practices adapted the implementation strategies to concentrate initially on various sub-groups e.g. patients with poorly controlled hypertension; those of working age who were more likely to find surgery visits difficult; low risk patients; or those assessed as being more able to manage the system. As a result, the non-participating patients (comparator group) were systematically different at baseline from those in the telemonitoring group.
(2) White coat effect	Surgery readings were likely to be affected by ‘white coat effect’ [9], whereby BP tends to be higher in clinical settings compared to home settings. This causes confounding bias when seeking to determine the difference between readings taken by the telemonitoring group at home and readings taken in the comparator group in the surgery.
(3) High variability in the frequency of readings	Surgery readings were often recorded much less frequently than home readings, raising the possibility of a type of ascertainment bias whereby raising or lowering of BP is much more easily identified for those in the telemonitoring group. This is related to the problem highlighted by Goldstein (2020) whereby data may be collected at different rates or not at all, and therefore any missing data may be informative of underlying health status [1]. Patients in the Scale-up BP study were on different protocols for how frequently they should measure their BP and (probably for clinical reasons) these varied over time. For example: less frequent protocols would be required when BP is stable than when adjusting treatment to improve control. Adherence may also have varied over time.
(4) Contamination of readings	We observed that home readings are sometimes transcribed by general practitioners into practice systems which are thus indistinguishable from surgery readings, [6] making comparison of apparent surgery readings prone to error. Although this was likely to have occurred more frequently in the telemonitoring group, it could also have occurred in the comparator group (e.g. if comparator patients had access to home reading systems). In any case, this would have had the effect of diluting the intervention effect.
(5) Regression to the mean	Reductions in BP over time were likely to be affected by regression-to-the mean in both groups, but particularly in the telemonitoring group as patients were prospectively selected for telemonitoring (perhaps based on their level of BP control).
(6) Measurement error	Individual BP readings will be measured with measurement error such that they may deviate from their true value. This is expected to increase the overall variability of BP measurements.
(7) End digit preference	We had discovered that a limited level of end digit bias was present in home telemonitored readings [10], however we were unsure as to how large this effect would be in surgery based readings.
(8) Withdrawal bias	A small proportion of patients (7%) dropped out of the telemonitoring arm, and there were missing data across both groups. We found that telemonitoring patients with higher starting systolic BP were more likely to drop out [6]. The reason for this is unknown.

We attempted to address as many of these issues as possible in our analysis by using four different approaches that are compared in this setting: (i) standardisation with stratification, (ii) standardisation with matching, (iii) regression adjustment for propensity score, and (iv) mixed-effects modelling. The issues faced and the methods we used to address these have broad applicability to other studies using routinely acquired data.

## Methods

### Data processing

Some processing of the raw BP data was necessary before analysis could proceed to exclude any erroneous observations. We applied the following exclusion criteria (which were the same as those used in our previous end digit preference study [10]):

- Systolic BP less than 60 mmHg
- Systolic BP greater than 262 mmHg
- Diastolic BP less than 40 mmHg
- Diastolic BP greater than 124 mmHg
- Diastolic BP greater than systolic BP
- Systolic BP less than 10 mmHg higher than diastolic

Patients who did not use the telemonitoring system were identified as comparator patients. To make the comparator patients as similar as possible to the telemonitoring patients, we only included patients between 18 and 90 years old, and excluded any surgery BP readings measured before telemonitoring was introduced in Lothian (1st Sept 2015).

### End digit preference

There is widespread evidence for end digit preference in BP measured by clinicians in the surgery or by patients at home using manual telemonitoring systems [10–13]. It is therefore recognised as an important source of potential bias in BP records. Although the occurrence and magnitude of end digit preference among the telemonitoring patients included in this study has already been thoroughly evaluated in another paper [10], we were uncertain about the magnitude of end digit preference in surgery measured BP values among comparator patients. We therefore sought to evaluate the extent of end digit preference in the comparator group and compare this with the results from our previous paper based on the telemonitored BP [10]. This involved using bar charts of end digits and a simple cross tabulation of systolic BP end digits against diastolic BP end digits to determine end digit frequencies and the prevalence of double-zero digit preference (i.e. both systolic BP and diastolic BP end with a zero). No formal hypothesis testing was performed because strongly significant *p*-values were highly likely due to the very large sample size.

### Standardisation with stratification

Differences in the frequency of readings between telemonitoring and comparator groups led us to try to standardise the comparison of “before” and “after” when analysing the change in BP readings over time. The frequency of readings was also highly variable within each group. We therefore calculated the change in BP values between baseline and a second reading 6–12 months later for all patients, where the second reading was taken to be as close as possible to 12 months if there was choice between multiple readings. For simplicity, we refer to this second reading 6–12 months later as a “final reading”. In the telemonitoring group; “baseline” was taken to be the second reading after the patient started using the telemonitoring system due to a concern that the first reading may have been used to test the system. For the comparator patients, they did not (to our knowledge) use the telemonitoring system and so it was important that we avoided using historical BP readings taken before the telemonitoring system was rolled out in Lothian to reduce the possibility of secular time-related biases. To that end, we only used “baseline” BP readings from comparator patients taken after 1st September 2015, the start of telemonitoring. Also, we only included patients with a full year of follow-up (e.g. those only recently recruited to telemonitoring were excluded). In the comparator group, any patients with age recorded as being under 18 or over 90 years old at the start of the telemonitoring service were excluded to make the groups as comparable as possible since no children or extremely elderly patients were recruited to use telemonitoring.

Descriptive statistics were calculated for the BP differences overall and stratified by important pre-specified subgroups. These were sex (male/female), age (< 65/65+), index systolic BP, and Scottish Index of Multiple Deprivation (SIMD) 2012 decile (< 5 / 5+) [14]. (For SIMD, lower values indicate a higher level of deprivation [14].) Stratification was important in this population, as the groups may have differed according to sociodemographic characteristics.

BP differences were calculated as baseline minus final reading. These differences were then compared between telemonitoring and comparator groups by fitting linear mixed effects models to the data, with BP difference as the outcome variable, and adjusting for SIMD (< 5 versus 5+), gender, and age. We then stratified according to systolic BP, rather than including this variable in the model, to avoid any bias due to modelling the relationship between change and initial value in regression models [15]. GP practice was included in the models as a random effect.

The percentage of patients with raised systolic and diastolic BP at baseline and follow-up (final reading 6–12 months later) were calculated for various thresholds indicating raised BP (135 + mmHg, 140 + mmHg, 145 + mmHg, and 150 + mmHg for systolic BP; 85 + mmHg and 90 + mmHg for diastolic BP) with percentage relative risk reductions presented for the change in BP over time in the telemonitoring and comparator groups. For the 145 + mmHg threshold comparison, we illustrate the use of a “relative risk reduction ratio” to compare between groups, with approximate bootstrap 95% confidence intervals calculated using the non-parametric bootstrap based on 9999 resamples.

### Standardisation with matched cohort analysis

Baseline and final BP values were calculated as for the stratified analysis. Telemonitoring patients were matched against comparator patients in a 1:1 ratio according to: (i) exact SIMD, (ii) gender, (iii) age by decade (e.g. 50s, 60s), and (iv) first systolic BP for comparator patients (and second systolic BP for telemonitoring patients) to the nearest value ending in 0 or 5 (e.g. systolic BP 130, 135, 140).

All surgery measurements from comparator patients (systolic BP and diastolic BP) were reduced by − 5 prior to analysis to take into account the expected ‘white coat effect’ and to attempt to make them more comparable with comparator patients. This difference is supported by the National Institute for Health and Care Excellence (NICE) guidelines for the diagnosis of hypertension [9]. Nevertheless, we tested this assumption in sensitivity analyses below.

After matching, final systolic and diastolic BP values were compared between the telemonitoring and comparator groups using a paired t-test. Only final values in the time window between six and 12 months after the index baseline BP reading were considered. As in the stratified analysis, no BP measurements prior to September 2015 were included. We also ensured that only independent matched pairs of patients were included in the analysis. If more than one comparator patient could be matched with a telemonitoring patient, then the comparator patient with BP measurements closest in time to the telemonitoring measurement was selected.

Practice effect was not adjusted for in the models: when we tried to adjust for practice effect as a random variable, the estimate of the variance was zero.

### Regression adjustment for propensity score

Propensity score methods aim to summarise a list of confounders into a single score where each propensity score represents the probability of group membership (intervention/control) for each subject based on a list of confounders. We applied a “regression adjustment for the propensity score” method [16] to the same standardized dataset as used in the previous two methods. An advantage of this method is that it still enables unbiased estimation of treatment effects in linear models conditional on confounders if only the propensity score model is correctly specified and not necessarily the outcome regression model [16].

To derive the propensity score, we first fitted a simple logistic regression model to the group variable (telemonitoring versus control), with SIMD 5+, female gender, patient age, and systolic BP at baseline as covariates. This model generated predicted values for group measurement for all individuals which served as the propensity scores. These propensity scores were then adjusted for in a separate linear mixed effects regression model fitted to final systolic BP with intervention group as an explanatory variable and conditioned on propensity score. GP practice was included as a random effect in the models.

### Random coefficient modelling

A random coefficients model (mixed effects analysis) was used to analyse the BP data for each practice and overall. Only BP outcome data collected after 1st September 2015 were included in the analysis, except that we adjusted for the number of surgery BP measurements prior to the start of telemonitoring (or prior to first surgery BP reading after 1st September 2015 in comparator patients). We did not place any time restriction on the data other than the 1st September 2015 cut-off, and so this method had the advantage of using all available surgery and telemonitoring BP data. Surgery BP measurements from telemonitoring patients were also included. The first telemonitored BP value recorded for each patient was deleted in case this had been used to test the system.

A random coefficients model was fitted to the BP outcome data for each practice, and each model included the following explanatory variables:

- Patient time indicating the number of weeks after the first telemonitoring or surgery BP measurement was recorded.
- Group indicator variable (0 = Surgery BP measurements from surgery patients, 1 = Surgery BP measurements from Telemonitoring patients, 2 = Telemonitoring BP measurements).
- Patient time and group interaction term
- Random intercept term for patient
- Random effect for patient time (random slope)
- Number of BP measurements recorded in the year prior to first measurement, as a categorical variable (0, 1–4, 5 or more)
- Approximate patient age in 2015 (based on year of birth), as a continuous linear term.
- Scottish Index of Multiple Deprivation (SIMD) of 5 or higher (yes or no)
- Sex (male or female)

Unstructured covariance was assumed.

In the model we considered within-patient time instead of calendar time because we were primarily interested in how BP changed over time *within patients* after they started using telemonitoring rather than changes over calendar time, which may have been confounded by systematic differences in the recruited population over time as practices rolled out the service.

Note that the “group indicator” main effect variable adjusts for systematic differences in baseline systolic BP and so this variable in theory should have taken full account of any potential ‘white coat effect’. The main focus of the results was on the patient time and group interaction term since we were interested in how changes in BP over time varied with treatment group.

The within-practice model results were then combined in a random-effects meta-analysis using the DerSimonian-Laird estimator. We used the “metafor” package [17] in R software [18]. The rationale for using a random-effects rather than a fixed-effect meta-analysis was that we were interested in generalising the results to all 128 practices in Lothian (not only the eight practices included in the evaluation). An overall analysis including data from all practices was possible, but at the cost of not being able to adjust for practice. An overall model including both practice and patient random-effects was fitted, but did not converge. We think this was because the model was trying to estimate a between-practice variability in outcome that was effectively zero (or close to zero), and so an overall model without the practice random-effects seemed reasonable.

### Ethical considerations

This study was approved by the East of England–Cambridge South Research Ethics Committee (16/EE/0058). We made use of several routine electronic health care data sources that were linked, de-identified, and held in the NHS Research Scotland (NRS) Lothian Research Safe Haven, only accessible by approved individuals who had undertaken the necessary governance training. Patients participating in telemonitoring provided individual written consent for their data to be analysed. Anonymised data from comparator patients in the same practices was unconsented. The local Caldecott Guardian gave permission for the anonymised data to be analysed within the NHS Safe Haven on the grounds of patient benefit. It was only possible to export analysis results from the NHS Safe Haven that avoided the identification of individual patients.

## Results

### Data processing

**Fig. 1:**
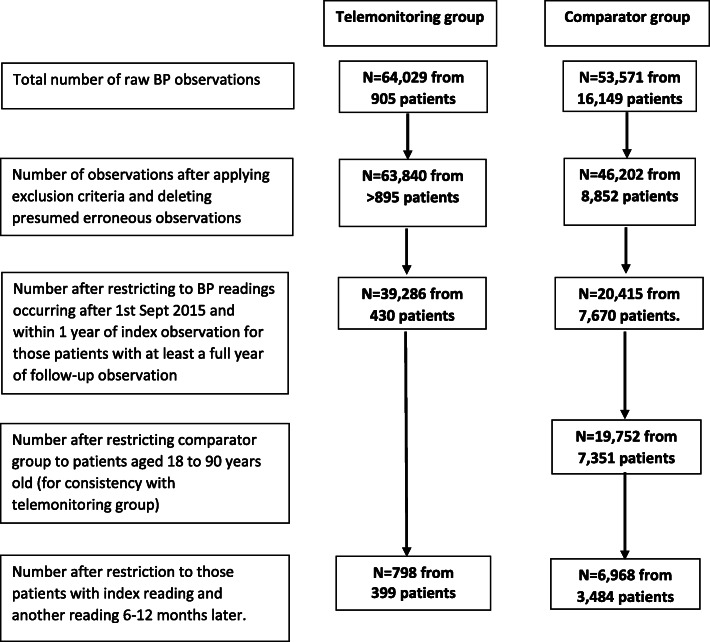
Summary flow diagram of data processing procedure

Figure 1 shows a summary flow diagram for the number of observations and patients at each stage of the processing procedure. In the raw telemonitoring dataset, there were 64,029 telemonitored BP observations from 905 patients, but this was reduced to 63,840 observations after applying the exclusion criteria and deleting presumed erroneous observations. Restricting to BP readings within 1 year of the index observation and patients with a least a full year of follow-up, the number of observations was reduced to 39,286 observations from 430 patients. After further restriction to those patients with a second Florence reading and another reading 6–12 months later, the number of patients reduced to 399.

In the raw database of comparator patients, there were 53,571 observations from 16,149 patients, and after applying the same exclusion criteria and restrictions as for the telemonitoring group, this number was reduced to 20,415 observations from 7670 patients (see Fig. 1). After further restriction involving deleting all patients under 18 or older than 90 years, and excluding any patients not recording first and last BP more than 6 months apart, the number of patients reduced to 3484.

### End digit preference

**Fig. 2:**
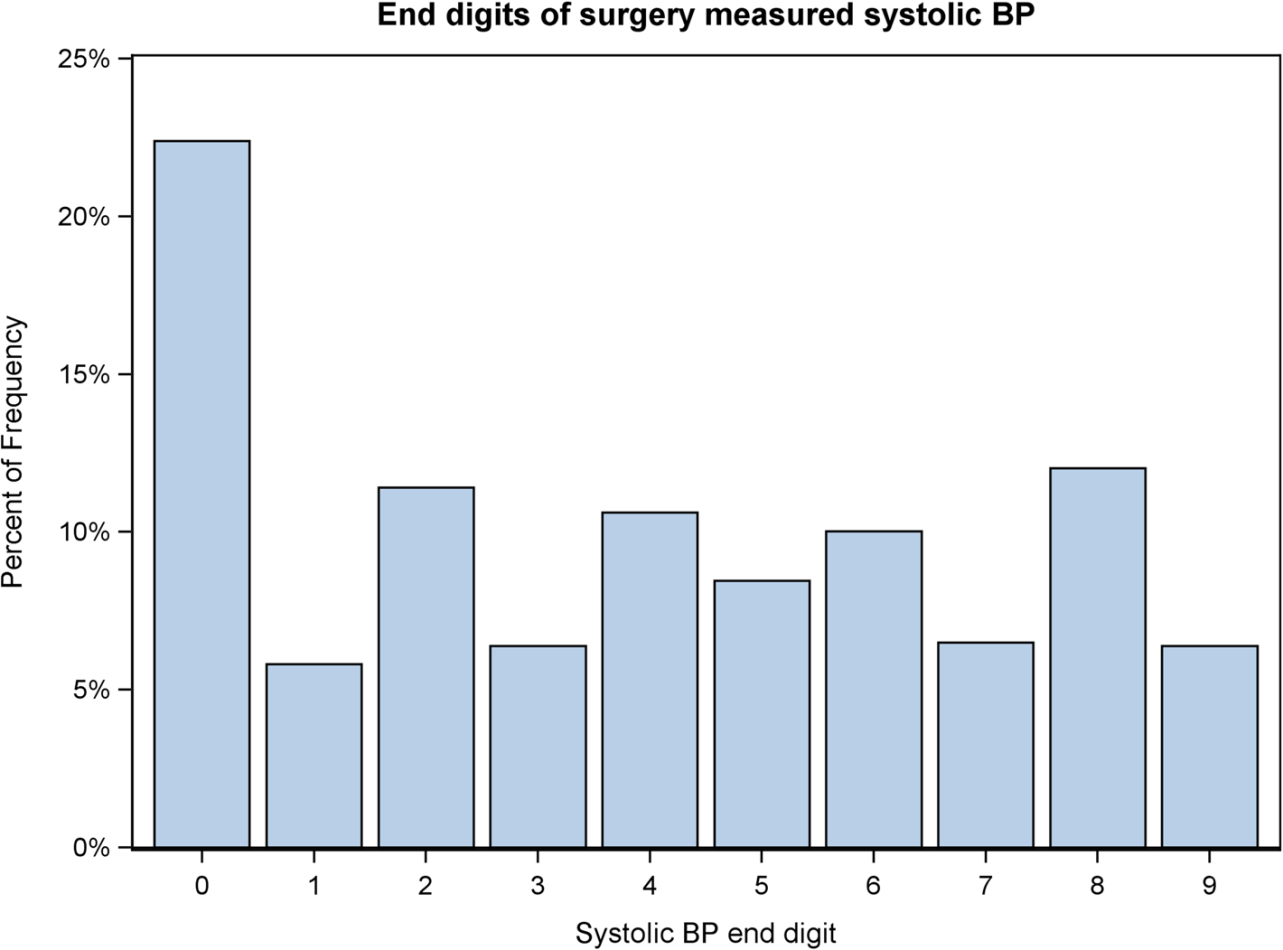
End digits of surgery measured systolic BP in comparator patients

A cross-tabulation of surgery measured systolic BP end digits against diastolic BP end digits is shown in Table S1 in the supplementary file. We observed a very strong double-zero preference in surgery-measured BP. The percentage of BP readings with double zeros was 11% (5877/54,073) which is much higher than the percentage expected by chance of 1% and the percentage of 1.7% (761/44,150) we observed in telemonitored BP readings [10]. For systolic BP individually, Fig. 2 shows a markedly higher percentage of BP readings ending with a zero, with a similar pattern being observed for diastolic BP (see Figure S1 in supplementary file). There is also a suggestion of a preference for even end digits since all odd digits are below the even digits in both bar charts.

### Standardisation with stratification

Table 2 shows patient characteristics for those patients in the telemonitoring and comparator groups who had at least two BPs 6–12 months apart, and with at least 1 year of follow-up. The follow-up duration was restricted to 12 months for all patients.

**Table 2:** Characteristics of patients in the telemonitoring and comparator groups, used for the stratification and matched analyses

	Telemonitoring (***n*** = 399)	Comparator group (***n*** = 3484)
Female sex	182/399 (46%)	1845/3484 (53%)
Age	Mean 62.5 (SD 9.7)	Mean 69.7 (SD 12.3)
	Median 64 (IQR 56 to 70)	Median 71 (IQR 62 to 79)
	Range 29 to 89	Range 20 to 90
SIMD 2012 decile	Mean 7.9 (SD 2.5)	Mean 7.0 (SD 12.3)
	Median 9 (IQR 6 to 10)	Median 7 (IQR 5 to 10)
	Range 2 to 10	Range 1 to 10
Index systolic BP reading^a^	Mean 139.6 (SD 16.4)	Mean 140.0 (SD 18.1)
	Median 138 (IQR 128 to 150)	Median 138 (IQR 130 to 150)
	Range 100 to 188	Range 71 to 240

^a^Index systolic BP values were unadjusted for white coat effect

Comparator patients were older on average, with a slightly higher percentage of females, and lower SIMD (i.e. more deprived). Index systolic BP readings were similar.

Table 3 shows the percentage of patients with raised systolic and diastolic BP at baseline and follow-up (final reading 6–12 months later) for the subgroup of patients with valid BP values at both baseline and follow-up.

**Table 3:** Percentage with raised SBP and DBP

	Telemonitoring			Comparator		
	Index reading	6–12 months later	Percentage Relative Risk reduction	Index reading	6–12 months later	Percentage Relative Risk reduction
SBP 135+	190/399 (48%)	94/399 (24%)	51%	2119/3484 (61%)	1879/3484 (54%)	11%
SBP 140+	138/399 (35%)	51/399 (13%)	63%	1658/3484 (48%)	1414/3484 (41%)	15%
SBP 145+	92/399 (23%)	37/399 (9%)	60%	1132/3484 (32%)	854/3484 (25%)	25%
SBP 150+	62/399 (16%)	20/399 (5%)	68%	894/3484 (26%)	555/3484 (16%)	38%
DBP 85+	138/399 (35%)	66/399 (17%)	52%	1080/3484 (31%)	799/3484 (23%)	26%
DBP 90+	90/399 (23%)	23/399 (6%)	74%	672/3484 (19%)	411/3484 (12%)	39%

*SBP* Systolic BP, *DBP* Diastolic BP

The observed improvements in BP control over time were larger in the telemonitoring group. For example, the percentage of patients with systolic BP of 145 mmHg or above was 14% lower at 6–12 months follow-up compared to baseline (relative risk reduction of 60% (95% CI 46 to 72)) for those in the telemonitoring group, compared to only 7% lower for comparator group patients (relative risk reduction of 25% (95% CI 19 to 29)). Therefore, the relative risk reduction in the telemonitoring group was more than double what it was in the comparator group (relative risk reduction ratio 2.43, 95% CI 1.77 to 3.27). Even after taking into account ‘white coat effect’ and comparing to those in the comparator arm with systolic BP of 150 + mmHg, the relative risk reduction was still greater in the telemonitoring arm (relative risk reduction ratio 1.58, 95% CI 1.17 to 2.00).

Table 4 shows descriptive statistics for the change in systolic BP (baseline – follow-up) for the telemonitoring group, with similar changes for the comparator group in brackets for comparison, stratified according to baseline variables. Note that no adjustment for ‘white coat effect’ has been made to the data in this table. Stratifying the results like this allowed us to see that the greatest differences in BP change between telemonitoring and comparator groups were for males, older patients (over 65 years), and those with relatively low systolic BP at baseline, although there may have been some confounding between each of these variables. A similar table for diastolic BP differences is shown in the supplementary file (Table S2).

**Table 4:** Systolic BP differences in mmHg (baseline – final readings)

Stratification	N	Mean	SD	Median	IQR	Range
**None (Overall)**	399 [3484]	6.5 [3.5]	15.2 [19.5]	6 [2]	−3 to 15 [− 8 to 14]	− 37 to 63 [− 87 to 88]
**Age < 65**	211 [1049]	6.4 [4.5]	14.6 [19.0]	6 [4]	− 3 to 16 [− 8 to 15]	−28 to 55 [− 65 to 88]
**Age 65+**	188 [2435]	6.7 [3.1]	15.8 [19.7]	6.5 [2]	−3.5 to 13.5 [−9 to 14]	− 37 to 63 [− 87 to 88]
**Male**	217 [1639]	6.9 [2.7]	15.2 [18.7]	7 [2]	−3 to 15 [−9 to 14]	− 37 to 63 [− 87 to 88]
**Female**	182 [1845]	6.1 [4.2]	15.2 [20.1]	5 [3]	−3 to 15 [−8 to 15]	− 34 to 53 [− 75 to 88]
**SIMD < 5 (more deprived)**	70 [811]	7.8 [4.1]	13.4 [19.2]	6.5 [3]	0 to 16 [−8 to 16]	−25 to 50 [−55 to 88]
**SIMD 5+ (more affluent)**	329 [2673]	6.3 [3.3]	15.5 [19.5]	6 [2]	−3 to 15 [−9 to 14]	− 37 to 63 [−87 to 88]
**Systolic BP < 135**	209 [1365]	−1.2 [−7.9]	11.8 [14.9]	0 [−7]	− 7 to 7 [− 16 to 2]	− 37 to 28 [− 72 to 44]
**Systolic BP 135 or above**	190 [2119]	15.1 [10.8]	13.9 [18.5]	13 [9]	6 to 23 [0 to 21]	−17 to 63 [−87 to 88]
**Systolic BP 140 or above**	138 [1658]	17.7 [13.6]	14.0 [18.8]	16.5 [12]	9 to 25 [2 to 24]	−17 to 63 [−75 to 88]
**Systolic BP 145 or above**	92 [1132]	20.9 [18.3]	14.0 [18.9]	21 [18]	11 to 27.5 [7 to 29]	−17 to 63 [−75 to 88]
**Systolic BP 150 or above**	62 [894]	23.8 [21.4]	14.8 [18.8]	22.5 [21]	12 to 34 [10 to 32]	−10 to 63 [−75 to 88]

Numbers are shown as Telemonitoring [Comparator]

We then fitted a linear mixed effects model in each strata of systolic BP. The results for the group variable (telemonitoring – comparator) are shown in Table S3. Note that all of these results occurred after applying a − 5 ‘white coat effect’ adjustment.

The improvement in BP control was significantly greater for telemonitoring patients compared to comparator patients for patients with systolic BP below 135 at baseline (4.06 (95% CI 1.82 to 6.30, *p* < 0.001), but no significant difference was observed in the other categories (see Table S3). Telemonitoring appears to have a protective effect against increased systolic BP over time in those with already fairly low systolic BP at baseline.

### Standardisation with matched cohort analysis

The mean difference in final systolic BP and diastolic BP (Comparator patients – Telemonitoring patients) were 5.96 (95% CI 3.55 to 8.36, *p* < 0.001) and − 0.10 (95% CI − 1.81 to 1.60, *p* = 0.904), respectively.

Therefore, the final systolic BP was lower for telemonitoring patients compared to comparator patients in matched analysis after 6–12 months, even after reducing the systolic BP of comparator patients by a − 5 ‘white coat effect’ adjustment.

We also performed detailed sensitivity analyses, adjusting the matching criteria, and also the amount we adjusted the surgery systolic BP readings (see Table 5).

**Table 5:** Sensitivity analyses for standardisation with matched analysis (Systolic BP)

	Matching criterion for Systolic BP	Adjustment to surgery Systolic BP readings^a^	N	Systolic BP		
				Mean difference	95% confidence interval	*P*-value
1	Nearest SBP with end digit 0 or 5	0	212	7.11	5.03 to 9.19	< 0.001
2	Nearest SBP with end digit 0 or 5	−7	201	4.01	1.47 to 6.56	0.002
3	Nearest SBP with end digit 0 or 5	−10	211	1.83	−0.55 to 4.21	0.131
4	Exact SBP matching	0	119	5.70	2.78 to 8.61	< 0.001
5	Exact SBP matching	−5	120	5.67	2.24 to 9.09	0.001
6	Exact SBP matching	−7	128	2.25	−0.67 to 5.17	0.130
7	Exact SBP matching	−10	123	2.23	−0.74 to 5.19	0.140
8	Nearest SBP with end digit 0	−5	208	3.90	1.39 to 6.42	0.003
9	Nearest SBP with end digit 0	−7	209	2.90	0.53 to 5.28	0.017

^a^Adjustment was applied to matching values as well as final values

The sensitivity analyses suggested that results were quite sensitive to our assumption about the ‘white coat effect’, although we note that reduction of the surgery systolic BP readings had to be quite large to overturn the result of a significant systolic BP difference in favour of telemonitoring. If no ‘white coat effect’ adjustment was made to diastolic BP, the mean difference was 3.07 (95% CI 1.43 to 4.71), which was also statistically significant. The sensitivity analyses for diastolic BP are shown in Table S4 in the Supplementary file.

### Regression adjustment for propensity score

Final systolic BP was significantly lower in the telemonitoring group after adjusting for the propensity score and assuming a − 5 adjustment for white coat effect (mean difference − 3.73, 95% CI − 5.34 to − 2.13, *p* < 0.0001). This difference remained, even after applying a − 7 adjustment (mean difference − 2.19, 95% CI − 3.80 to − 0.58, *p* = 0.01).

### Random coefficients model analysis

The random coefficients model analysis had the advantage of using all the BP outcome data for patients as well as being able to take into account the time of measurements after each patient first started using telemonitoring (or first started recording readings after September 2015 in the comparator group). Table 6 shows the patient characteristics of this sample.

**Table 6:** Patient characteristics of all patients in the telemonitoring and comparator groups

	Telemonitoring (***n*** = 882)	Comparator group (***n*** = 7806)
Female sex	413/882 (47%)	4115/7806 (53%)
Age	Mean 62.5 (SD 10.2)	Mean 68.7 (SD 12.7)
	Median 64 (IQR 56 to 70)	Median 70 (IQR 60 to 79)
	Range 22 to 89	Range 19 to 90
SIMD 2012 decile	Mean 7.7 (SD 2.5)	Mean 7.0 (SD 2.5)
	Median 8 (IQR 6 to 10)	Median 7 (IQR 5 to 10)
	Range 2 to 10	Range 1 to 10
Index systolic BP reading	Mean 134.4 (SD 16.4)	Mean 140.1 (SD 18.2)
	Median 134 (IQR 124 to 144)	Median 139 (IQR 129 to 150)
	Range 90 to 205	Range 71 to 240

As Table 2 showed, comparator patients were older on average, with a slightly higher percentage of females, and lower SIMD. Interestingly, unlike in Table 2 which showed no clear difference, baseline systolic BP was higher among the comparator patients on average compared to the telemonitoring group.

**Fig. 3:**
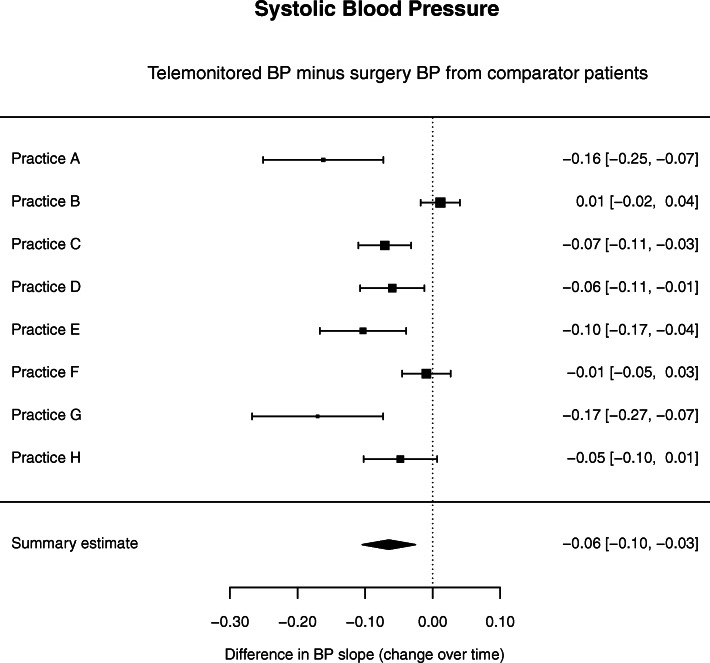
Forest plot showing between-group differences in change of systolic BP for telemonitored BP – surgery measured BP in comparator patients

Figure 3 shows the mean differences of systolic BP change per week (with 95% confidence intervals) for telemonitored BP in telemonitoring patients versus surgery measured BP in comparator patients in each practice, with a summary effect size computed using random effects meta-analysis.

Systolic BP change over time was significantly higher in the telemonitored group. The weekly improvement under telemonitoring was estimated to be − 0.06 (95% CI − 0.10 to − 0.03) or − 3.37 (95% CI − 5.41 to − 1.33) per year. The overall analysis across all sites, unadjusted for site, gave a very similar result of − 0.06 (95% CI − 0.08 to − 0.04) or − 3.19 (− 4.16 to − 2.23) per year, albeit more precise.

Note that by means of the group main effect term in the random coefficients model this analysis adjusts for ‘white coat effect’, provided that the magnitude of this potential bias remained constant over time, which is a plausible assumption.

The figures show high variation in results across practices with a few practices (especially small practices) showing large effects of telemonitoring.

Figure S2 in the supplementary file shows a similar plot for change in diastolic BP.

Additionally, Figures S3 and S4 show forest plots for the comparison of surgery measured BP between telemonitoring and comparator patients for systolic and diastolic BP respectively, but due to widespread entry of telemonitored readings into GP surgery systems these results should be interpreted with caution.

### Overall assessment of analyses

In Table 7, we consider how well all of the analyses address the biases outlined in the Introduction section. All analyses were conducted using SAS software version 9.4 (SAS Institute Inc., Cary, NC, USA) except where indicated above.

**Table 7:** Assessment of how well the analyses control for key potential biases

Challenges	Ability of the models to control for bias
(1) Non-randomised design	Limited. Stratification helps to some extent to control confounding, but covariate adjustment in linear mixed effects models provides better adjustment. There still may have been residual confounders. For the matched cohort analysis, there was excellent control of confounding due to matching, but there still may have been residual confounding by underlying variables not used to match on.
(2) White coat effect	Depends on the validity of the assumption for the difference due to ‘white coat effect’. Matched cohort analysis results were found to be fairly sensitive to this assumption. In the random coefficients model, this was fully adjusted by means of adjusting for intervention/control group at baseline, although we make the reasonable assumption that the degree of ‘white coat effect’ did not change over time.
(3) High variability in the frequency of readings	Partially. Standardisation meant that frequencies of readings were the same between groups, but subgroup selection to achieve this may have resulted in a biased subgroup. Regression adjustment for propensity score or matching may have only partially addressed this bias by controlling for confounders between groups. Mixed effects models make a missing-at-random assumption for missing data. If this assumption holds true in estimating the change in BP over time, then the difference in frequency of readings would have had no effect on the estimated treatment effect because the change in BP would be correctly modelled in each group. However, if the reason for missing data (or different frequencies) was more or less informative in one of the groups compared to the other however (e.g. indicating low BP in comparator patients) then this could have biased the results.
(4) Contamination of readings	Not an issue. All methods compared telemonitored BP with surgery measured BP from comparator patients.
(5) Regression to the mean	At least partially. Will be controlled to some extent due to comparison with comparator group and matching, but there may be differences in the strength of regression-to-the-mean between treatment and comparator groups. For example, it is conceivable that between-group differences in the inclusion probabilities for patients with greater propensity for stronger regression-to-the mean (e.g. those with intermittently high or unstable BP), might contribute to confounding bias.
(6) Measurement error	This was not addressed by any of the analysis methods based on the standardised data. We expect that there might have been attenuation of the intervention effect towards zero as a result. The random coefficients model did not fully address this, although the model did include all of the multiple BP measurements per patient which would have improved estimation of within-patient variability and the underlying true change in BP within each patient.
(7) End digit preference	End digit preference will compound the effect of any measurement error such that it will lead to observed values deviating from their true values. The analyses were limited in their ability to deal with end digit preference in the same way as for measurement error. If there was differential change in end digit preference or specific value preference over time in one group compared to the other, then it may have caused confounding bias. For the matched analysis, patients may not have been matched correctly by systolic BP due to differential end digit bias between groups. Again, we were reliant on the reliability of the assumption about the true BP in each group. Adjustment for group at baseline in a random coefficient model should in theory have adjusted for differences in the strength of digit preference.
(8) Withdrawal bias	For the analyses based on the standardised dataset we used subgroup selection to select out everyone with at least two readings at baseline and follow-up. Patients who withdrew from the telemonitoring arm or those in the comparator arm who got their BP measured less frequently were more likely to be excluded from the analysis, and so this problem reduces to the problem of incomparable groups and residual confounding (issue (1)). For the random coefficient model analysis, this analysis assumes any missing data is “missing-at-random” conditional on covariates used in the adjustment. If the reasons for missing data or missing data mechanisms differed according to treatment group, and these were not taken into account in the statistical model, then this may have biased the results.

## Discussion

This implementation study provides further supportive evidence to suggest that improved BP control due to telemonitoring seen in previous RCTs is also present when rolling out telemonitoring in the community. We previously reported a reduction in systolic BP over time for telemonitoring patients, but a key limitation was that it may have been affected by regression to the mean [6]. This study is therefore a step forward because it compares the telemonitoring BPs to a contemporaneous comparator group. However, although we adjusted for key variables at baseline, at the analysis stage we could not completely exclude residual differences between the cohorts at baseline. We also faced a number of other challenges described in this paper such as substantial differences in the frequency of BP readings between cohorts, unrepresentative surgery measured BPs (e.g. due to ‘white coat effect’), differential levels of end digit preference, and erroneous or missing data. End digit preference in particular was higher in the comparator group compared to the telemonitoring group. The percentage of BP readings with double zeros was 11%; which although higher than the 1.7% we observed for telemonitored readings in our previous study [10], was still lower than what was found in a recent study by Greiver et al. [11], which suggested a value of approximately 17% [11]. This could indicate greater use of automated sphygmomanometers in the participating GP surgeries [11] or greater use of self-purchased home monitors by some patients in the comparator group (assuming home readings were then transcribed by GPs).

Many of the challenges and potential biases we have encountered will also be relevant for other studies using routinely acquired data. Indeed, some of these challenges have already been reported in other studies [1, 19].

The random coefficients model analysis had the advantage of using all the data, and of the four methods was thought to have controlled best for the ‘white coat effect’. The other methods relied on assumptions about the degree of ‘white coat effect’ that may not have been true. However, the random coefficients analysis was still susceptible to residual confounding and changes in the level of biases over time (including changes in ‘white coat effect’). Differences in the frequency of readings between groups may also have caused an issue due to missing data bias. The random coefficient models assumed a linear change in BP over time, which increased interpretability of the results and appeared to be supported by line plots over 12 months, but this was still an assumption that may have masked underlying non-linearity in changes over time. Adjustment of potential confounders may also have been improved by fitting splines; but we did not do this due to the risk of convergence failure. Indeed, we experienced problems fitting random-effects for both practice and patient. The models did not converge, which may have been because the between practice effect was close to zero. Fitting separate per practice models and then combining in a random effects meta-analysis helped us to circumvent the problem of the model failing to converge when the practice variable was in the model. The forest plots also allowed us to compare results across the different practices. Indeed, it was interesting to observe substantial variation across practices, with some small practices showing strong telemonitoring effects. This is not surprising given that practices had different policies for introducing the telemonitoring, with some focussing on uncontrolled patients while others on well controlled.

Matched cohort studies are a useful way to eliminate known confounders [20]. In this study, we matched on (i) exact SIMD, (ii) gender, (iii) age in decades (e.g. 50, 60), and (iv) index systolic BP to the nearest value ending in 0 or 5. After matching we used a paired t-test for analysis rather than a linear mixed regression model adjusting for the matching variables to maximise the precision of estimation. This is a valid approach since we did not adjust for additional confounders; but, as for the other analyses, this approach also assumes that there were no additional confounders or other sources of bias [21]. Although this analysis provided the greatest control over baseline covariates through matching, the sample size of available matched pairs was fairly small and many sensitivity analyses were required. Propensity score matching was an alternative method which may have improved the numbers of matched pairs. This analysis method has been widely used in practice over the last 30 years to control for selection bias in observational studies [22]. An advantage of the method is that it only requires matching on a single variable (the propensity score), rather than matching on multiple covariates, and so is easier to use [22]. Indeed, it is particularly useful if the number of covariates available for matching is large [22]. Although this approach has received some criticism in recent years, it is an appropriate method if used with care [22, 23]. In particular, there is a need to check the balance of key prognostic factors across intervention/control groups after matching [22].

We applied a different propensity score method involving “regression adjustment for propensity score”, recognising the advantage that propensity score methods have in terms of being able to summarise a long list of confounders into a single score [16]. In our case, we only had routinely collected data on a few confounders so this advantage could not be fully realised. Nevertheless, the method enabled unbiased estimation of treatment effect even in the case of an incorrectly specified outcome regression model, provided that the propensity score model was correctly specified [16]. In addition, the method allowed us to make use of all data available from the standardized dataset, unlike the matched cohort analysis.

The “standardisation with stratification” method had the advantage of highlighting subgroups in which the between group differences were greatest. The results suggested that older patients or those with lower levels of hypertension showed particular benefit from using telemonitoring, implying that telemonitoring systems should not only be restricted to younger patients (who might be perceived to be more technically literate) or those with very high levels of hypertension, but should be offered widely to those on the hypertension register. Indeed this finding provides some evidence for persistent BP monitoring rather than just titration to control and stopping.

A strength of this study was that four different statistical methods were used to analyse the data and reached similar conclusions, although none of the methods could completely exclude the possibility of residual confounding and all methods were susceptible to changes in certain biases over time (e.g. level of ‘white coat effect’, transcription of home readings into GP practice systems, and differential changes in end digit preference). For studies involving routinely acquired data in general, it is important that researchers are aware of these potential biases and consider in advance how their statistical analyses will address these. Applying multiple statistical methods to the same problem gives reassurance that any results observed are not dependent on the statistical method, although as we have seen there may be some overlap in their methodological limitations.

A limitation of all our analyses was that we did not take into account potential measurement error in the blood pressure outcomes. Instead of using single measurements at baseline and 6–12 months later, we could have calculated the average of three (or more) readings at baseline and 6–12 months which would have reduced within-patient variability. However, this would have substantially reduced the overall sample size for all of the analyses because many patients recorded fewer than three readings at baseline and 6–12 months later. The impact of any measurement error and/or end digit rounding bias is expected to attenuate the intervention effect towards zero. The fact that we observed a telemonitoring effect across all methods despite the possibility of measurement error bias or end digit bias, only serves to strengthen our conclusion of a real telemonitoring effect.

We also recognise that comparing the random coefficient analysis method against the other methods was not really a fair comparison in some respects because this method was the only method that utilised all of the available data. However, we believe that precisely because of this reason, the random coefficient analysis should be recommended above others in this context, due to its potential to give more representative and generalizable results in this pragmatic study. Although the other analyses based on the standardized dataset may have ensured that the telemonitoring/control groups were more consistent in terms of their frequency of BP measurement, these analyses may still have been affected by residual selection bias. For the random coefficients analysis, there was a large sample size available of over 7500, with only a few confounders available to adjust for. In other settings with smaller sample size and a greater number of confounders, it may be more advantageous to adjust for the propensity score in the random coefficients analysis to increase statistical power, especially if there is missing data on some covariates.

This was a real world roll-out of a telehealth intervention which had the advantage that patients did not need to sign up to the research or attend clinic visits for data collections. Other designs (especially novel randomised trials) may have provided better control of biases, but they were likely to have been less pragmatic or achievable in this setting and the trial processes may have led to reduced external validity. A cluster randomised trial design was explored as a potential study design, although for clinically relevant “hard” outcomes such as stroke or ischaemic heart disease, these outcomes are rare and so the sample size requirement was extremely high. For example, we previously calculated we would need 25,643 patients per group (51,286 in total), in order to detect a relative risk reduction of 15% with 90% power, assuming a two-sided 5% significance level; and that is even before applying an inflation factor to take into account potential withdrawals or to allow for clustering by practice. Note also, that many of the biases may still have been present even in a randomised design (e.g. differential changes in end digit preference). Finally, we acknowledge that the duration of follow-up for many of our patients was short (up to 12 months). Longer term studies are appropriate to investigate if the telemonitoring effect continues or wanes over time.

Studies of telehealth interventions face the same trade-off between internal and external validity as studies of digital health interventions more generally [24, 25]. That is, studies with high internal validity such as randomized controlled trials of telemonitoring are likely to have limited external validity due to rigorous trial procedures, increased face-to-face contact between research staff and participants, and by inclusion of a motivated consent-to-trial population [25]. Indeed, there is often a danger that randomised trial processes constitute an intervention in their own right and thereby increase adherence and patient motivation [26]. On the other hand, non-randomised implementation studies are better for informing public policy due to improved external validity and generalisability, but with the greater potential for various biases affecting the results. We therefore recommend that both types of studies are conducted to provide a wide-ranging evidence base, but the challenge is to develop statistical methods capable of addressing the complex array of biases that may be present in implementation studies, particularly in those involving routinely acquired data. As always, the context of study is crucial. Not all of the biases we have listed in this article will be relevant for every implementation study; even in those studies comparing BP using routinely acquired data. However, we hope that the list of biases we have provided can help as a useful starting point in implementation studies involving routinely acquired data. At the design stage, we recommend that researchers collect data on as many potential confounders as possible prior to analysis and adjust for them in the analysis model either individually or via propensity score. Researchers should be wary of how any biases will influence the analysis results and adjust the strength of their conclusions accordingly.

## Conclusions

In conclusion, our study provides additional evidence of the effectiveness of telemonitoring, and suggests that initiatives to roll out telemonitoring at scale should be encouraged. Future implementation studies are needed to confirm the findings of our study, particularly those enabling longer-term follow-up. Routine data give us the opportunity to monitor if expected improvements in outcome occur, but appreciation of potential biases and careful development of analytical methods is important to ensure that the findings are reliable. The random coefficient analysis is particularly recommended in this setting due to its ability to utilise all available data and take into account multiple repeated measurements per patient.

## Supplementary Information

**Additional file 1: Table S1.** End digits for systolic BP against end digits for diastolic BP (count and %). **Table S2.** Diastolic BP differences in mmHg (baseline – final readings). **Table S3.** Linear mixed effects model results for systolic BP reduction. **Table S4.** Sensitivity analyses for matching analysis of diastolic BP. **Figure S1.** End digits of surgery measured diastolic BP in comparator patients. **Figure S2.** Forest plot showing between-group differences in change of diastolic BP for telemonitored BP – surgery measured BP in comparator patients**. Figure S3.** Forest plot showing between-group differences in change of systolic BP for surgery measured BP (telemonitoring – comparator). **Figure S4.** Forest plot showing between-group differences in change of diastolic BP for surgery measured BP (telemonitoring – comparator).

## Data Availability

Scale-up BP made use of several routine electronic health care data sources that are linked, de-identified, and held in the NHS Research Scotland (NRS) Lothian Research Safe Haven, which is only accessible by approved individuals who have undertaken the necessary governance training. Therefore, the datasets generated and/or analysed during the current study are not publicly available to maintain patient confidentiality and prevent the identification of individual patients.

## Abbreviations

BP: Blood pressure
DBP: Diastolic Blood Pressure
HITS: Health Impact of nurse-led Telemetry Services
NHS: National Health Service
NRS: National Health Service Research Scotland
RCT: Randomised Controlled Trial
SBP: Systolic Blood Pressure
SIMD: Scottish Index of Multiple Deprivation
TEC: Technology Enabled Care
