# Supplementary material for: Examining the effectiveness of telemonitoring with routinely acquired blood pressure data in primary care: challenges in the statistical analysis
Source: BMC Med Res Methodol. 2021 Feb 10;21:31. doi: 10.1186/s12874-021-01219-8 (PMC7877114; doi:10.1186/s12874-021-01219-8)

**Table S1:** End digits for systolic BP against end digits for diastolic BP (count and %)

| **End digit of systolic BP** | **End digit of diastolic BP** | | | | | | | | | | |
| --- | --- | --- | --- | --- | --- | --- | --- | --- | --- | --- | --- |
|  | **0** | **1** | **2** | **3** | **4** | **5** | **6** | **7** | **8** | **9** | **Total** |
| **0** | \| 5877 \| \| --- \| \| 10.87 \| | \| 384 \| \| --- \| \| 0.71 \| | \| 1030 \| \| --- \| \| 1.90 \| | \| 351 \| \| --- \| \| 0.65 \| | \| 980 \| \| --- \| \| 1.81 \| | \| 840 \| \| --- \| \| 1.55 \| | \| 736 \| \| --- \| \| 1.36 \| | \| 365 \| \| --- \| \| 0.68 \| | \| 1137 \| \| --- \| \| 2.10 \| | \| 409 \| \| --- \| \| 0.76 \| | \| 12109 \| \| --- \| \| 22.39 \| |
| **1** | \| 367 \| \| --- \| \| 0.68 \| | \| 346 \| \| --- \| \| 0.64 \| | \| 314 \| \| --- \| \| 0.58 \| | \| 283 \| \| --- \| \| 0.52 \| | \| 332 \| \| --- \| \| 0.61 \| | \| 251 \| \| --- \| \| 0.46 \| | \| 341 \| \| --- \| \| 0.63 \| | \| 279 \| \| --- \| \| 0.52 \| | \| 333 \| \| --- \| \| 0.62 \| | \| 293 \| \| --- \| \| 0.54 \| | \| 3139 \| \| --- \| \| 5.81 \| |
| **2** | \| 1334 \| \| --- \| \| 2.47 \| | \| 357 \| \| --- \| \| 0.66 \| | \| 916 \| \| --- \| \| 1.69 \| | \| 315 \| \| --- \| \| 0.58 \| | \| 748 \| \| --- \| \| 1.38 \| | \| 402 \| \| --- \| \| 0.74 \| | \| 606 \| \| --- \| \| 1.12 \| | \| 376 \| \| --- \| \| 0.70 \| | \| 811 \| \| --- \| \| 1.50 \| | \| 307 \| \| --- \| \| 0.57 \| | \| 6172 \| \| --- \| \| 11.41 \| |
| **3** | \| 379 \| \| --- \| \| 0.70 \| | \| 326 \| \| --- \| \| 0.60 \| | \| 333 \| \| --- \| \| 0.62 \| | \| 337 \| \| --- \| \| 0.62 \| | \| 337 \| \| --- \| \| 0.62 \| | \| 338 \| \| --- \| \| 0.63 \| | \| 334 \| \| --- \| \| 0.62 \| | \| 347 \| \| --- \| \| 0.64 \| | \| 370 \| \| --- \| \| 0.68 \| | \| 353 \| \| --- \| \| 0.65 \| | \| 3454 \| \| --- \| \| 6.39 \| |
| **4** | \| 1166 \| \| --- \| \| 2.16 \| | \| 291 \| \| --- \| \| 0.54 \| | \| 662 \| \| --- \| \| 1.22 \| | \| 347 \| \| --- \| \| 0.64 \| | \| 844 \| \| --- \| \| 1.56 \| | \| 351 \| \| --- \| \| 0.65 \| | \| 592 \| \| --- \| \| 1.09 \| | \| 363 \| \| --- \| \| 0.67 \| | \| 758 \| \| --- \| \| 1.40 \| | \| 365 \| \| --- \| \| 0.68 \| | \| 5739 \| \| --- \| \| 10.61 \| |
| **5** | \| 972 \| \| --- \| \| 1.80 \| | \| 327 \| \| --- \| \| 0.60 \| | \| 388 \| \| --- \| \| 0.72 \| | \| 318 \| \| --- \| \| 0.59 \| | \| 372 \| \| --- \| \| 0.69 \| | \| 689 \| \| --- \| \| 1.27 \| | \| 380 \| \| --- \| \| 0.70 \| | \| 329 \| \| --- \| \| 0.61 \| | \| 423 \| \| --- \| \| 0.78 \| | \| 367 \| \| --- \| \| 0.68 \| | \| 4565 \| \| --- \| \| 8.44 \| |
| **6** | \| 1000 \| \| --- \| \| 1.85 \| | \| 343 \| \| --- \| \| 0.63 \| | \| 646 \| \| --- \| \| 1.19 \| | \| 342 \| \| --- \| \| 0.63 \| | \| 644 \| \| --- \| \| 1.19 \| | \| 363 \| \| --- \| \| 0.67 \| | \| 680 \| \| --- \| \| 1.26 \| | \| 386 \| \| --- \| \| 0.71 \| | \| 688 \| \| --- \| \| 1.27 \| | \| 330 \| \| --- \| \| 0.61 \| | \| 5422 \| \| --- \| \| 10.03 \| |
| **7** | \| 443 \| \| --- \| \| 0.82 \| | \| 314 \| \| --- \| \| 0.58 \| | \| 341 \| \| --- \| \| 0.63 \| | \| 303 \| \| --- \| \| 0.56 \| | \| 395 \| \| --- \| \| 0.73 \| | \| 303 \| \| --- \| \| 0.56 \| | \| 325 \| \| --- \| \| 0.60 \| | \| 387 \| \| --- \| \| 0.72 \| | \| 384 \| \| --- \| \| 0.71 \| | \| 318 \| \| --- \| \| 0.59 \| | \| 3513 \| \| --- \| \| 6.50 \| |
| **8** | \| 1431 \| \| --- \| \| 2.65 \| | \| 324 \| \| --- \| \| 0.60 \| | \| 799 \| \| --- \| \| 1.48 \| | \| 386 \| \| --- \| \| 0.71 \| | \| 718 \| \| --- \| \| 1.33 \| | \| 416 \| \| --- \| \| 0.77 \| | \| 654 \| \| --- \| \| 1.21 \| | \| 336 \| \| --- \| \| 0.62 \| | \| 1035 \| \| --- \| \| 1.91 \| | \| 404 \| \| --- \| \| 0.75 \| | \| 6503 \| \| --- \| \| 12.03 \| |
| **9** | \| 431 \| \| --- \| \| 0.80 \| | \| 305 \| \| --- \| \| 0.56 \| | \| 361 \| \| --- \| \| 0.67 \| | \| 309 \| \| --- \| \| 0.57 \| | \| 348 \| \| --- \| \| 0.64 \| | \| 322 \| \| --- \| \| 0.60 \| | \| 314 \| \| --- \| \| 0.58 \| | \| 314 \| \| --- \| \| 0.58 \| | \| 396 \| \| --- \| \| 0.73 \| | \| 357 \| \| --- \| \| 0.66 \| | \| 3457 \| \| --- \| \| 6.39 \| |
| **Total** | \| 13400 \| \| --- \| \| 24.78 \| | \| 3317 \| \| --- \| \| 6.13 \| | \| 5790 \| \| --- \| \| 10.71 \| | \| 3291 \| \| --- \| \| 6.09 \| | \| 5718 \| \| --- \| \| 10.57 \| | \| 4275 \| \| --- \| \| 7.91 \| | \| 4962 \| \| --- \| \| 9.18 \| | \| 3482 \| \| --- \| \| 6.44 \| | \| 6335 \| \| --- \| \| 11.72 \| | \| 3503 \| \| --- \| \| 6.48 \| | \| 54073 \| \| --- \| \| 100.00 \| |

**Table S2: Diastolic BP differences in mmHg (baseline – final readings)**

| **Stratification** | **N** | **Mean** | **SD** | **Median** | **IQR** | **Range** |
| --- | --- | --- | --- | --- | --- | --- |
| **None (Overall)** | 399  [3484] | 4.23  [1.88] | 8.68  [11.61] | 4  [1] | -1 to 10  [-6 to 10] | -24 to 29  [-44 to 51] |
|  |  |  |  |  |  |  |
| **Age <65** | 211  [1049] | 3.94  [3.16] | 8.36  [12.13] | 3  [2] | -1 to 10  [-4 to 11] | -22 to 26  [-36 to 44] |
| **Age 65+** | 188  [2435] | 4.55  [1.33] | 9.03  [11.34] | 4.5  [1] | -1 to 11  [-6 to 8] | -24 to 29  [-44 to 51] |
| **Male** | 217  [1639] | 4.25  [1.45] | 8.77  [11.69] | 4  [1] | -1 to 10  [-6 to 9] | -24 to 29  [-40 to 41] |
| **Female** | 182  [1845] | 4.19  [2.27] | 8.60  [11.53] | 4  [1] | -1 to 10  [-5 to 10] | -19 to 26  [-44 to 51] |
| **SIMD<5 (more deprived)** | 70  [811] | 3.69  [2.26] | 8.23  [11.85] | 2.5  [2] | -2 to 9  [-6 to 10] | -14 to 26  [-44 to 47] |
| **SIMD 5+ (more affluent)** | 329  [2673] | 4.34  [1.77] | 8.78  [11.54] | 4  [1] | -1 to 10  [-6 to 9] | -24 to 29  [-40 to 51] |
| **SBP<135** | 209  [1365] | 1.45  [-2.21] | 7.47  [10.07] | 1  [-2] | -3 to 7  [-9 to 4] | -21 to 23  [-40 to 33] |
| **SBP 135 or above** | 190  [2119] | 7.28  [4.52] | 8.91  [11.78] | 8  [4] | 1 to 13  [-3 to 12] | -24 to 29  [-44 to 51] |
| **SBP 140 or above** | 138  [1658] | 8.38  [5.48] | 8.70  [11.97] | 8  [5] | 3 to 14  [-2 to 13] | -24 to 29  [-44 to 51] |
| **SBP 145 or above** | 92  [1132] | 9.83  [7.36] | 8.37  [12.31] | 10  [8] | 4 to 15.5  [0 to 15] | -16 to 29  [-44 to 51] |
| **SBP 150 or above** | 62  [894] | 11.06  [8.47] | 8.86  [12.32] | 12  [8.5] | 6 to 17  [0 to 17] | -16 to 29  [-40 to 51] |

Numbers are shown as Telemonitoring [Comparator]

**Table S3: Linear mixed effects model results for systolic BP reduction**

|  | **Coefficient** | **95% CI** | **P-value** |
| --- | --- | --- | --- |
| Patients with systolic BP<135 only | 4.064 | 1.825 to 6.302 | 0.0004 |
| Patients with systolic BP>=135 only | 1.494 | -1.391 to 4.379 | 0.310 |
| systolic BP>=135 and Age>=65 | 3.150 | -0.979 to 7.278 | 0.135 |
| Patients with systolic BP>=140 only | -0.287 | -3.697 to 3.124 | 0.869 |
| Patients with systolic BP>=145 only | -0.150 | -4.271 to 3.971 | 0.943 |
| Patients with systolic BP>=150 only | -1.633 | -6.500 to 3.235 | 0.510 |

**Table S4: Sensitivity analyses for matching analysis of diastolic BP**

|  | **Matching criterion for Systolic BP** | **Adjustment to surgery Systolic BP readings*** | **N** | **Diastolic BP** | | | |
| --- | --- | --- | --- | --- | --- | --- | --- |
|  |  |  |  | **Mean difference** | **95% confidence interval** | **P-value** |  |
| 1 | Nearest SBP with end digit 0 or 5 | 0 | 212 | 3.07 | 1.43 to 4.71 | <0.001 |  |
| 2 | Nearest SBP with end digit 0 or 5 | -7 | 201 | 0.01 | -1.76 to 1.79 | 0.987 |  |
| 3 | Nearest SBP with end digit 0 or 5 | -10 | 211 | -0.02 | -1.62 to 1.58 | 0.981 |  |
| 4 | Exact SBP matching | 0 | 119 | 3.55 | 1.41 to 5.70 | 0.001 |  |
| 5 | Exact SBP matching | -5 | 120 | -1.68 | -3.96 to 0.59 | 0.146 |  |
| 6 | Exact SBP matching | -7 | 128 | -0.96 | -3.15 to 1.23 | 0.386 |  |
| 7 | Exact SBP matching | -10 | 123 | 1.28 | -0.85 to 3.42 | 0.236 |  |
| 8 | Nearest SBP with end digit 0 | -5 | 208 | -0.61 | -2.34 to 1.12 | 0.486 |  |
| 9 | Nearest SBP with end digit 0 | -7 | 209 | -0.68 | -2.37 to 1.02 | 0.430 |  |

*Adjustment was applied to matching values as well as final values. Final surgery DBP was consistently adjusted by -5 except in the analyses with no adjustment to SBP, in which case the adjustment to final DBP was also 0.

**Figure S1: End digits of surgery measured diastolic BP in comparator patients**


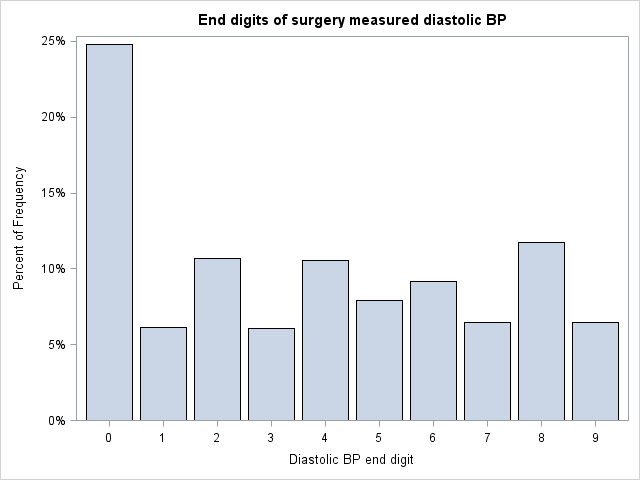


**Figure S2: Forest plot showing between-group differences in change of diastolic BP for telemonitored BP – surgery measured BP in comparator patients.**


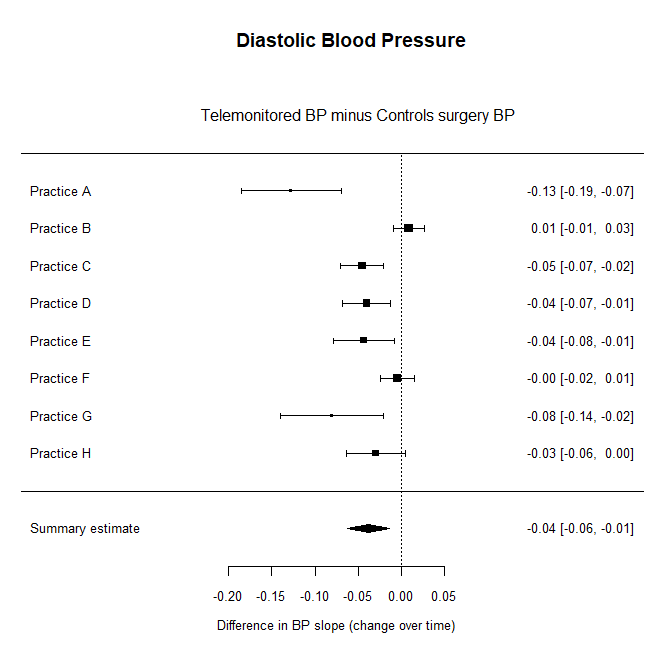


**Figure S3: Forest plot showing between-group differences in change of systolic BP for surgery measured BP (telemonitoring – comparator)**


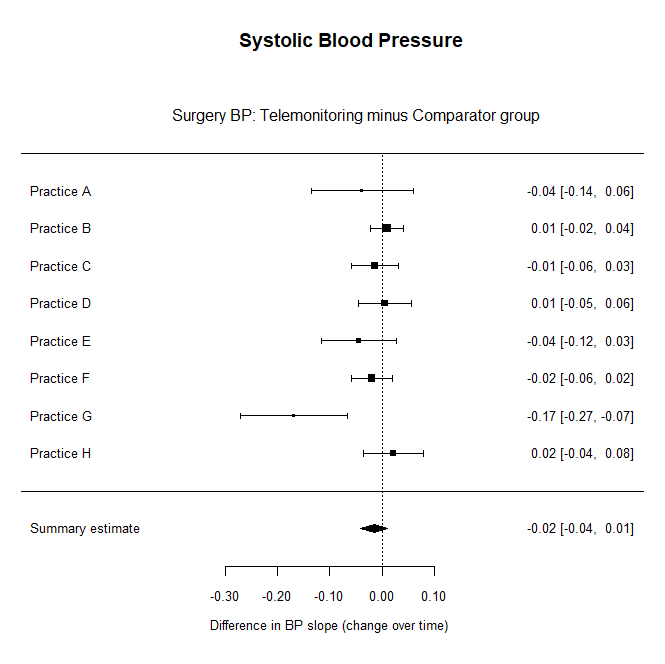


**Figure S4: Forest plot showing between-group differences in change of diastolic BP for surgery measured BP (telemonitoring – comparator)**


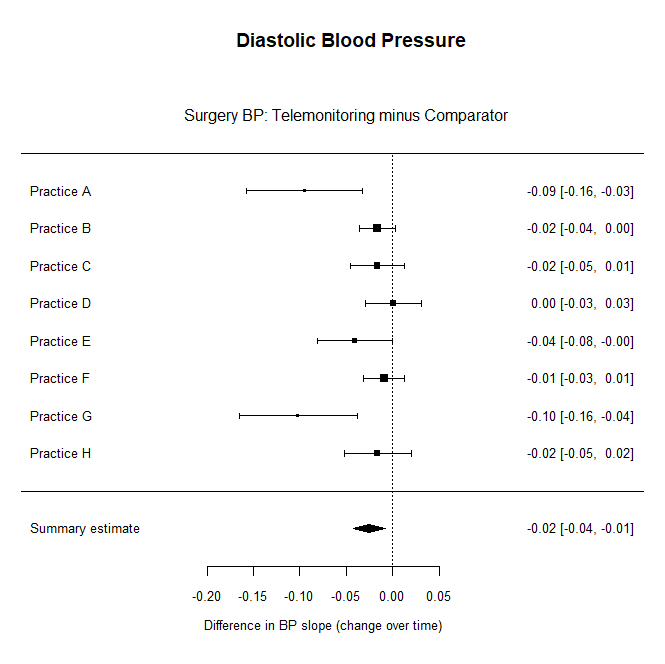

Supplement: Supplementary file 1 — Additional file 1: Table S1. End digits for systolic BP against end digits for diastolic BP (count and %). Table S2. Diastolic BP differences in mmHg (baseline – final readings). Table S3. Linear mixed effects model results for systolic BP reduction. Table S4. Sensitivity analyses for matching analysis of diastolic BP. Figure S1. End digits of surgery measured diastolic BP in comparator patients. Figure S2. Forest plot showing between-group differences in change of diastolic BP for telemonitored BP – surgery measured BP in comparator patients. Figure S3. Forest plot showing between-group differences in change of systolic BP for surgery measured BP (telemonitoring – comparator). Figure S4. Forest plot showing between-group differences in change of diastolic BP for surgery measured BP (telemonitoring – comparator). [file 12874_2021_1219_MOESM1_ESM.docx]
